# Dr. Addison Goes

**Published:** 1921-04-09

**Authors:** 


### THE PUBLIC WELL-BEING.

### Dr. Addison Goes.

### THE TASK OF HIS SUCCESSOR.

The removal of Dr. Addison from the position of Minister of Health must be a matter for regret to all sincere believers in the cause of public health. Few public men running a great Department of State have been more vilified; yet few have worked with greater assiduity and singleness of purpose to master a series of baffling problems that many would have refused to face. Dr. Addison had a supremely difficult task, because in the nature of the case, it was impossible for him to frame any policy upon any aspect of public health without incurring obloquy from some interested quarter. A man who tries to supervise, control, and develop on progressive lines a national scheme of public health is bound to "interfere" with some privilege or cherished tradition of active interest, and thus it was that Dr. Addison was very rarely out of "hot water." His fault was not that he did too little, but that in honest endeavour he tried to do too much. The one charge that his numerous and varied critics were able to level with justice against his ill-fated omnibus Bill was that it covered a field of such extraordinary expanse that only a limited number of experts were able to discern its total value or to disentangle its complex and overburdened provisions. With regard to housing, with which question Dr. Addison's name is chiefly associated in the minds of the lay public, we think the ex-Minister has been most unjustly assailed. For many months practically the entire responsibility for the failure of the housing schemes in this country was angrily ascribed to him. But if he had been the spirit of wisdom and knowledge incarnate we do not see how Dr. Addison could have overcome the fundamental difficulties of scarce and costly material, scarce money, and scarcer labour.

His task was gigantic. In the earlier days of the problem it can now be seen that it was beyond the power of the State even partially to meet the public demand for houses. Yet Dr. Addison is entitled to the gratitude of the public—though probably he does not expect to get it—for the courage and pertinacity with which he applied himself to the task, and for his resourcefulness in experiment and expedients. Those who have been most intimately associated with the ex-Minister know that he kept an open and receptive mind and was always ready to consider new ideas from any quarter. The housing situation is already easier, and in the near future it will probably be easier still if the Government boldly adhere to their scheme for the dilution of building labour, without regard to the selfish attitude of the trade unions concerned. To that extent Dr. Addison's successor, Sir Alfred Mond, will benefit from the pioneer work already done by an energetic Ministry. Nobody, however, will be inclined to envy Sir Alfred Mond his legacy in the broader region of public health. The rejected Bill, as we have frequently pointed out, will have to be reintroduced in some form, and whether it is presented to Parliament in a single large dose or in several small doses, it is bound to arouse once more a storm of opposition, especially at a time when the cry for economy is universal and loud. It is of little use, we are afraid, to point out to the angry and indiscriminate opponents of "squandermania" that the service of public health in this country is relatively and absolutely the cheapest service of all. The reformers will probably have to bow their heads before the storm until it has passed. But the time will come when every class in the nation will be compelled to recognise the sheer truth of the doctrine that what is called economy in public health is merely the most dangerous of all forms of national waste.

